# Relay-Assisted D2D Transmission for Mobile Health Applications

**DOI:** 10.3390/s18124417

**Published:** 2018-12-13

**Authors:** Hongcheng Huang, Wei Xiang, Yang Tao, Biao Liu, Min Hu

**Affiliations:** 1 School of Communication and Information Engineering, Chongqing University of Posts and Telecommunications, Chongqing 400065, China; s170131014@stu.cqupt.edu.cn (W.X.); s160131095@stu.cqupt.edu.cn (B.L.); humin@cqupt.edu.cn (M.H.); 2 Chongqing Engineering Research Center of Communication Software, Chongqing 400065, China

**Keywords:** Device-to-Device (D2D), mobile health, relay, full-duplex communication, trust

## Abstract

Relay-assisted Device-to-Device (D2D) communication, one of the important transmission modes in mobile health systems, can provide high transmission quality for servicing users at the edge of system coverage. However, the quality of the D2D relay communication is largely limited by the relay nodes. When a poor node is selected to assist the source node in the data transmission, it is likely to result in the loss of medical data and inaccurate transmission. Therefore, this paper focuses on how to select relay modes and relay nodes to improve the reliability of medical data transmission. Firstly, in order to eliminate the relay nodes with low energy or poor willingness, the acceptable energy consumption metric of relay nodes is proposed in this paper. The relay mode of each relay node is determined by the acceptable energy consumption metric, which can ensure the physical reliability of the relay communication links. Then a trust metric is proposed to measure the social reliability of each relay link, further excluding the malicious relay nodes. Finally, this paper proposes a relay selection algorithm based on compromise factors (RSCF). With the help of the proposed algorithm, the reliability of the relay communication can be guaranteed, and the spectrum efficiency can be promoted greatly. The simulation results show that the relay nodes selected by RSCF algorithm can greatly improve transmission rate and reliability compared with the traditional relay-assisted D2D communication schemes.

## 1. Introduction

With increased people’s interest in their own health, mobile health has gradually received widespread attention due to the capability of monitoring the physical conditions of healthy/diseased users in real time. Considering the privacy of medical data, most studies have focused on decreasing the eavesdropping probability of medical data during transmission by using encryption protocols or changing the medical delivery architecture [1,2]. However, in the mobile health system, communication modes have a great influence on medical data transmission, which is the basis of the entire mobile health system. For instance, the transmission reliability of relay communication itself is lower than that of the cellular communication. If a bad node is selected as a relay node in the relay communication, the medical data may not be correctly transmitted to the medical center or some data may even be lost. Thus, it is worthy to study the influence on the reliability of medical data transmission under different transmission modes. Moreover, all users in the mobile health system need to transmit information to the medical center through their own smart devices. Along with the continuous improvement and advancement of the mobile medical system, large-traffic applications such as online video diagnosis will gradually become popular. The ubiquitous biological information and various large-traffic medical data impose a great burden on the existing cellular system. Thus, it is worthy to consider how to offload the traffic of base stations and increase the spectrum utilization.

Device-to-Device (D2D) communication, a short-range communication technology, has great advantages in improving spectrum utilization of system and mitigating the base station’s load [3]. The D2D communication technology can directly multiplex the frequency resources of the cellular devices in the collaboration of base stations, thereby improving the spectrum utilization of the system. Furthermore, D2D communication can allow the direct data transmission among user devices without the assistance of base station. The signaling overhead of base stations and the number of repeated data transmissions can be reduced by D2D communication technology. Therefore, the traffic of base stations can be further offloaded. For the users, the user’s device can communicate with each other by a lower transmit power owing to the short-distance characteristic of D2D communication, thereby reducing the energy consumption of the user device. With the advantages of high spectrum utilization, low latency and low power consumption, D2D communication mode will obviously become an indispensable part in the future medical data transmission. In order to improve the transmission quality of user equipment at the edge of the cell, the relay-assisted D2D communication has also become one of the research hotspots. The relay-assisted D2D communication not only expands the communication distance, but also has the same advantages of high speed, low power consumption as D2D communication. However, when different nodes are selected to assist the source user in transmitting data, the quality of the relay communication may vary greatly. Each node in the system is selfish and limited by its own energy resources. Nodes with low energy and selfishness are likely to cause data loss or transmission interruptions, so it is extremely important to fully exploit the attributes and willingness of nodes. In summary, in order to ensure the reliability of medical transmission and effectively reduce the burden on the base stations, this paper discusses the influence of the system’s transmission modes on the medical data transmission, and focuses on the selection of both relay modes and relay nodes in D2D relay communication.

Consider the scenario of urban community where a medical center station is placed. The medical center station is equivalent to an edge node set by the hospital in each cell, which can uniformly manage various medical data of the cell, and provide corresponding services according to the request of the users. Users in the community wear various sensing devices (such as smart watches and smart glasses) to obtain all kinds of body sign data. The functions of the user’s smart device are as follows: (1) The user’s various medical data can be periodically transmitted to the edge medical node for storage and further analysis; (2) User equipment can directly connect to the edge medical node to request a diagnosis services, and the edge medical node also judges whether to provide online video diagnosis service according to the uploaded data; (3) For patients, when multiple physical data are not normal, the edge medical node will send early warning and guidance to the smart devices. In this scenario, the user’s device is considered as a hybrid full/half-duplex D2D communication device. The edge medical node, an edge node, can directly communicate with the user equipment. If users being far away from the edge node adopt the D2D straight-through communication mode, the transmission quality may not meet the needs of the transmission task. Therefore, the transmission quality can be improved through the relay-assisted D2D communication. Since the multi-hop D2D communication requires multiple relay nodes to forward data in turn, data will not be transmitted to the destination node if any relay node is interrupted. Considering that the possibility of data loss is high in multi-hop relay communication, this paper only discusses D2D relay communication with only two hops.

With the consideration of the privacy of medical data, how to ensure the transmission reliability is the key to this paper. The transmission reliability of D2D straight-through communication between fixed edge node and user devices is easy to be controlled. However, some users being farther away from the medical node need to D2D relay communication to improve the transmission quality, and the reliability of relay nodes is difficult to be controlled. Moreover, the frequent service requests of many users exert a great burden on the existing cellular system. In order to solve the above problems, this paper takes into account the equipment condition of relay nodes and the trust level of relay nodes. A relay selection mechanism based on the compromise factor is also designed. The contribution of this article is as follows:

- The selection of communication modes is determined by the relative distance between users and the edge medical node. In view of the relatively unstable relay communication in communication modes, the acceptable energy consumption metric of each relay node is proposed to exclude the relay nodes with weak willingness or insufficient energy. The duplex mode of each relay node is defined by the acceptable energy consumption and relay’s energy consumption.
- Considering the need of high reliability in medical data transmission, this paper evaluates the trust value of each relay node. The trust metric is divided into the average accuracy in previous relay communications and the possibility of generating D2D direct links. It is used to exclude the malicious relay nodes and comprehensive evaluate the reliability of relay nodes.
- In order to effectively measure the possibility of generating D2D direct links, this paper also proposes a relative distance stability value, which can analyze the frequency and duration of the distance among users less than the maximum D2D communication distance.
- Considering the varying importance of medical data, a relay node selection mechanism based on compromise factor is proposed. Through this mechanism, the maximum transmission rate can be prioritized under certain conditions.

The rest of this paper is organized as follows: Section 2 is about related work. Section 3 builds system patterns and problem descriptions. Section 4 proposes the method of relay pattern selection. In Section 5, the method of trust evaluation between the source node and the relay node is introduced in detail. Section 6 proposes relay selection mechanism based on compromise factor. Simulation results and performance analysis are presented in Section 7. Finally, a brief conclusion is provided in Section 8.

## 2. Related Work 

The mobile health system can monitor the user’s physical condition at any time and send emergency signals in a crisis, which is considered a reliable way to effectively improve the medical treatment quality and ensure user’s health. In the existing medical systems, the user’s medical data is transmitted to the medical center via cellular communication [4,5]. However, with the development of technology and the increasing limitation of spectrum resources, the transmission of mobile medical data will not be constrained by cellular communication in the future. D2D communication has great advantages in traffic offloading and controllability, thus it undoubtedly becomes an important transmission tool in the medical field [6]. Niraj et al. [7] applied D2D communication to mobile health systems. However, they focused on data exchange among multiple sensors in the body’s local area network. In [3], the authors considered that the normal operation of medical equipment can be affected by the electromagnetic interference of D2D and cellular communication, so a power control scheme is proposed to reduce the electromagnetic interference. D2D communication, as a kind of wireless communication method, is vulnerable to security attacks such as eavesdropping, fake messages, and privacy violations. Therefore, a secure-D2D data transmission protocol is proposed to improve transmission security [8]. Since the possibility of data loss and link interruption is small in D2D communication, the above studies only concentrated on improving the protocol security index in D2D communication or changing the medical transmission architecture. However, the quality of D2D communication is susceptible to distance limitations. If the D2D user’s channel quality is poor, it is extremely easy for the probability of eavesdropping and loss of medical data to increase.

In cellular systems, the channel quality is usually related to the user’s own device and the geographical location. For instance, the channel quality in cell edge is generally not good. In order to ensure the transmission quality of cell-edge users and further increase the system capacity, the D2D relay communication becomes a better solution. Melki et al. [9] compared and evaluated the performances of traditional cellular communication, one-hope D2D communication and relay-assisted D2D communication. The final simulation results showed that the D2D relay communication effectively improves system’s transmission quality and increases the system capacity. In D2D relay communication, the quality of the selected relay nodes greatly affects the system’s transmission efficiency and stability. Therefore, with the consideration of both the non-ideal interference environment and the global power limitation, a relay selection mechanism that maximizes the system transmission rate is proposed in [10]. In addition, the remaining power of the user equipment also affects the user’s willingness to be a relay node. Thus, the authors in [11] propose a cross-multilayer relay selection method via combining the time delay and the remaining power of the users. The simulation results show that the selected relay nodes have good overall performance. The above-described relay selection methods are all applied to D2D half-duplex relay communication, and do not consider D2D full-duplex relay communication that can greatly increase system capacity and reduce transmission delay.

In the D2D full-duplex relay communication, the D2D relay node can receive data from the source node and transmit cached data to the destination node simultaneously on the same time/frequency resource. Hence, the spectrum efficiency and transmission rate of the D2D full-duplex relay communication are about twice that of the half-duplex under ideal conditions. However, the performances of the D2D full-duplex relay communication are affected by self-interference, so how to reduce self-interference has received great attention [12,13,14]. In the existing studies of the relay-assisted D2D communication, many scholars have separately discussed the HD relay communication mode and the FD relay communication mode. Little work has been done to study the hybrid FD/HD relay communication. The authors of [15] compared the energy efficiency of FD and HD relay communications in the case of the controllable self-interference and the fixed power of source nodes. The results showed that the energy efficiency of FD relay communication is obviously better than that of HD relay communication. In [16], a unified model was proposed to represent full-duplex and half-duplex communication, and the model was used to simplify the process of mode selection and power distribution solution based on the maximum total transmission rate. In [17], an adaptive HD/FD selection scheme is proposed to maximize system capacity. The above researches selecting HD/FD mode are to maximize certain efficiency of the system, which do not measure whether the relay node can accept the selected duplex mode. Different relay nodes have different willingness and capacity of transmission. The selection of duplex modes only considers maximizing the system throughput or energy efficiency, which is difficult to ensure reliability in transmission. Therefore, according to the residual energy ratio and the willingness value of the relay node, this paper determines the relay node’s acceptable transmission mode and eliminates some relay nodes with insufficient transmission capability.

As the terminal equipment is carried by the user, the social relationship and mobility among the users have a great influence on the data transmission. Therefore, it is a research hotspot to integrate the influence of social attributes in the social network into the physical network [18,19,20,21]. In general, the higher the social relationship between the relay node and the source user, the stronger the stability of the relay transmission. In [22], a relay selection mechanism based on social factors is proposed, in which the transmission power of relay user is proportional to the social intensity between the source user and relay user, and the relay node is selected by minimizing interrupt probability. In [23], the user’s social behaviors are used to dynamically detect better relay nodes and the existing relay nodes can be changed by the better nodes at any time, thereby improving the stability of D2D relay communication. To obtain nodes with high trust, nodes’ behavioral characteristics are measured by the historical behaviors of the users [24,25]. Although the node willingness and transmission stability can be affected by the social domain to a certain extent, the strength of social relation itself is difficult to define or express. Therefore, how to define the strength of social relation has always been a problem worth studying. In [26], the social influence of the user is determined by fitting the similarity of the requested content among users and exploring user’s potential interest group in online social network. Zhang et al. [27] highlighted the influence of the mobility, which takes the predicted contact time among users as a part of measuring relationship strength. Based on the above measurement of social relationship, it is difficult to evaluate whether the relay node itself is a trustworthy node, such as a malicious node. Moreover, in order to obtain better communication quality, the number of selectable relay nodes has been limited to a certain extent. If the stability of D2D relay communication is measured only by considering the social relationship strength based on above methods, it is possible to exclude good strange nodes that always performed well in previous D2D relay communications. Based on the above problems, the trust metric is proposed in this paper, which takes into account the historical performance of relay nodes and the transmission possibility between the relay node and the source node to eliminate the malicious and low-reliability relay nodes.

## 3. System Model and Problem Description

### 3.1. System Model

**Figure 1:**
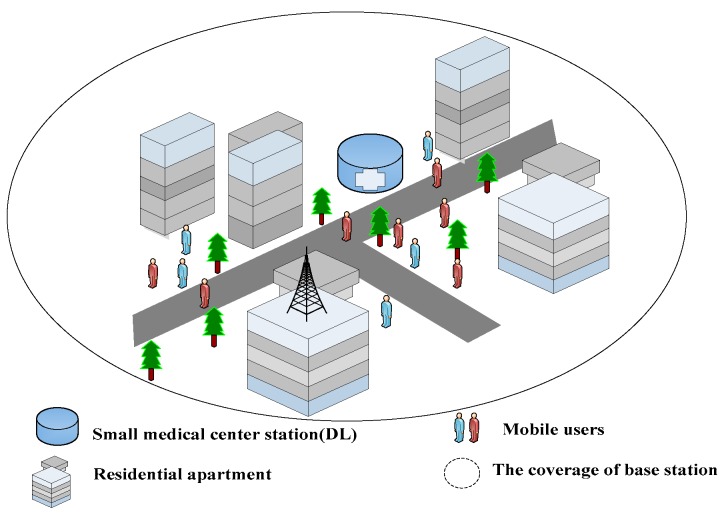
Medical scene graph in a single cell.

Consider a single cell scenario, where large numbers of user devices need to transmit medical data to an edge medical node. The base station as a mobile switching center controls the transmission modes and the power of wireless devices within its coverage. An edge medical node is placed in each cell and users are randomly distributed in the system. The specific scene diagram is shown in Figure 1.

In this system, the edge medical node is denoted as *DL*, and the users who need to transmit medical data at the current time are put in set *Q*. The set *C* refers to the cellular users set, and the remaining users set is *R*. 

Coordinates of base station is the origin, the position coordinates of any user equipment *u_i_* and edge medical node *DL* within the its coverage can be represented as (*r_i_,θ_i_*) and (*r_DL_,θ_DL_*), respectively. *r* and *θ* respectively represent the distance and the phase declination of the base station to the device. Therefore, the distance $D_{u_{i}}^{DL}$ from any user *u_i_* to the edge node *DL* can be expressed as:

(1) $$D_{u_{i}}^{D_{L}}=\sqrt{\left|{r_{i}}^{2}+{r_{DL}}^{2}-2r_{i}r_{DL}\cos(\theta_{i}-\theta_{DL})\right|}$$

Similarly, the distance $D_{u_{j}}^{u_{i}}$ between user *u_i_* and user *u_j_* can be expressed according to Equation (2):

(2) $$D_{uj}^{ui}=\sqrt{\left|{r_{ui}}^{2}+{r_{uj}}^{2}-2r_{ui}r_{uj}\cos(\theta_{ui}-\theta_{uj})\right|}$$

**Figure 2:**
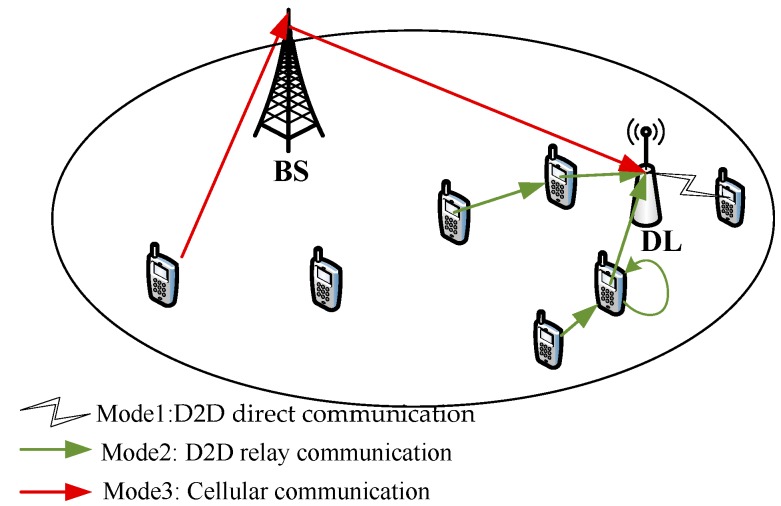
Communication mode selection schematic diagram based on distance between users and edge medical node.

Assuming that the transmission power of mobile devices is constant, the greater the transmission distance, the greater the attenuation of the signal. In order to ensure transmission quality, the transmission rate of the single-hop D2D communication cannot be lower than the minimum rate threshold, that is, the distance of any two D2D devices cannot be greater than the maximum communication distance $R_{U}$. According to the maximum communication distance and the geographic location of communication devices, the communication mode of each device in the cell can be roughly classified into the following three categories by only considering the path fading, as shown in Figure 2.

*Mode 1*: *D2D direct communication*. When $D_{u_{i}\in N}^{DL}<R_{U}$, the distance from the user who needs to transmit medical data to the edge node *DL* is in line with the single-hop D2D communication. In this case, the user equipment (UE) can directly communicate with the edge node by D2D communication. It not only saves UE’s energy consumption but also guarantees the quality of data transmission.

*Mode 2*: *D2D relay communication*. Since the multi-hop D2D communication requires multiple relay nodes to forward data in turn, if any relay node is interrupted, data will not be transmitted to the destination node, resulting in data loss. In order to reduce the possibility of data loss in multi-hop D2D communication, the data is mainly transmitted in the form of opportunistic broadcasting in the existing researches [28,29,30], that is, data transfer of each time needs broadcast to multiple users around, greatly increasing the possibility of data leakage. In summary, considering the sensitivity of medical data, this paper only considers two-hop D2D relay communication with only one relay node. Since the maximum transmission distance of the source node is $R_{U}$, the transmission power of relay nodes is generally not greater than the transmission power of the source device, that is, the maximum transmission radius of relay nodes is also $R_{U}$. Therefore, when the distance from users and the edge node satisfies the condition $R_{U}<D_{u_{i}\in N}^{DL}<2R_{U}$, the communication mode between the UEs and the edge node *DL* can adopt the two-hop D2D communication to improve the transmission performance.

*Mode 3*: *Cellular communication*. When $D_{u_{i}\in N}^{DL}\geq 2R_{U}$, it indicates that the distance form users to the edge node *DL* is relatively long. Considering that medical data pays more attention to transmission stability than video popular files, this paper does not consider multi-hop D2D communication. Therefore, cellular communication is used directly to transmit medical data.

### 3.2. Problem Description

According to the relative geographical location of users and edge node, the communication modes are divided into three categories, in which the D2D direct communication and the cellular communication between the user and the edge node *DL* are very stable transmission modes, and the stability of the link in the transmission process is determined by the quality of its own channel. However, relay-assist D2D communication requires relay equipment to cooperate for data transmission. The transmission quality for different relay nodes may be different, besides, the relay nodes also have the hybrid HD/FD communication capabilities. Therefore, the choice of both relay nodes and relay modes is the key to improve transmission quality and reliability.

## 4. Physical Network and the Acceptable Transmission Mode 

### 4.1. Physical Network of Relay Communication

The source nodes set requiring two-hop D2D relay communication can be obtained by the judgment of *Mode 2*. Let *sk* denote one of the source nodes, and the **Z***_sk_* is the candidate D2D relay set for source node *sk*. The selection criteria for any relay node *di* in the **Z***_sk_* can be denoted as:

(3) $$D_{sk}^{di}\leq R_{U}\;and\;D_{di}^{DL}\leq R_{U}$$

It is assumed that all channels obey independent Rayleigh fading, *h_i.j_* represents the channel gain among node *i* and node *j*, and obeys the exponential distribution of parameter *λ_i,j_*. In order to improve the frequency utilization, the source nodes and the relay nodes can multiplex the uplink channel resources of the cellular devices, in which the same-frequency interference value is expressed as *I*. All relay nodes apply the decode-and-forward (DF) method. Therefore, in HD relay communication, the $SINR_{sk\to di}^{HD}$ received by relay node *di* and $SINR_{di\to DL}^{HD}$ between the relay node *di* and the edge node *DL* are respectively represented as:

(4) $$SINR_{sk\to di}^{HD}=\frac{P_{sk}g_{sk,di}{\left|h_{sk,di}\right|}^{2}}{N_{0}+I_{c,di}}$$

(5) $$SINR_{di\to DL}^{HD}=\frac{P_{di}^{HD}g_{di,DL}{\left|h_{di,DL}\right|}^{2}}{N_{0}+I_{c,DL}}$$

where *g* represents the transmission path loss, *N*_0_ is the power of Gaussian white noise with 0 mean and *σ^2^* variance. *I_c,di_* indicates the same-frequency interference of the multiplexed cellular device *c* to the relay node *di*. Similarly, *I_c,DL_*. indicates the same-frequency interference of the multiplexed cellular user *c* to the edge node *DL*. *P_sk_* represents the transmission power of the source node *sk*. The transmission power of the relay node *di* is $P_{di}^{HD}$ satisfied by $P_{th-di}^{HD}\leq P_{di}^{HD}$. $P_{th-di}^{HD}$ represents the power threshold that satisfies the minimum transmission quality of the source user. Therefore, the value of $P_{th-di}^{HD}$ can be expressed as:

(6) $$P_{th-di}^{HD}=\frac{SINR_{th}\left(N_{0}+I_{c,DL}\right)}{g_{di,DL}{\left|h_{di,DL}\right|}^{2}}$$

*SINR_th_* represents a threshold for ensuring that the data of source node *sk* can correctly parse at the edge node *D_L_*. The source node does not reduce its own transmission power like the relay node due to its own energy limitation and selfishness. Therefore, the source node threshold is not considered in this paper and the transmission power of the source node is set to a constant value.

According to Equations (4) and (5), the total transmission rate $R_{sum\to di}^{HD}$ in HD relay communication mode can be expressed as:

(7) $$R_{sum\to di}^{HD}=\frac{1}{2}w_{c}\min\left(\log_{2}\left(1+SINR_{sk\to di}^{HD}\right),\log_{2}\left(1+SINR_{di\to DL}^{HD}\right)\right)$$

where *w_c_* indicates the spectrum bandwidth of the multiplexed cellular device.

Similarly, in FD relay communication mode, the $SINR_{sk\to di}^{FD}$ received by relay node *di* and $SINR_{di\to DL}^{FD}$ between the relay node *di* and the edge node *DL* are respectively represented as:

(8) $$SINR_{sk\to di}^{FD}=\frac{P_{sk}g_{sk,di}{\left|h_{sk,di}\right|}^{2}}{N_{0}+I_{c,di}+P_{di}^{FD}\Delta_{di}}$$

(9) $$SINR_{di\to DL}^{FD}=\frac{P_{di}^{FD}g_{di,DL}{\left|h_{di,DL}\right|}^{2}}{I_{c,DL}+N_{0}}$$

In Equation (8), $P_{di}^{FD}\Delta_{di}$ indicates that the relay node *di* is interfered by itself forwarding the signal. The value of $\Delta_{di}$ is [0,1], which indicates the ability of the relay node *di* to resist self-interference. The stronger the anti-interference ability, the larger the value of $\Delta_{di}$. $P_{di}^{FD}$ indicates the transmission power of the relay device in the full-duplex mode. Similarly, to ensure the quality of the FD relay communication, the power $P_{di}^{FD}$ needs to satisfy $P_{th-di}^{FD}\leq P_{di}^{FD}$. The value of $P_{th-di}^{FD}$ is:

(10) $$P_{th-di}^{FD}=\frac{SINR_{th}\left(N_{0}+I_{c,DL}\right)}{g_{di,DL}{\left|h_{di,DL}\right|}^{2}}$$

Based on Equations (8) and (9), the total transmission rate $R_{sum\to di}^{FD}$ in FD relay communication mode can be expressed as:

(11) $$R_{sum\to di}^{FD}=w_{c}\min\left(\log_{2}\left(1+SINR_{sk\to di}^{FD}\right),\log_{2}\left(1+SINR_{di\to DL}^{FD}\right)\right)$$

It can be seen from Equations (7) and (11) that the transmission rate of full-duplex relay communication is nearly twice that of half-duplex in the case of controllable self-interference [16]. However, when the transmission power is constant, the energy consumption of relay node in the full-duplex relay communication is significantly higher than that in the half-duplex relay communication. If the selected relay node does not consider the self energy consumption in full-duplex communication mode, it is difficult for the relay node to guarantee the transmission quality.

### 4.2. Relay Transmission Mode Selection Based on Acceptable Energy Consumption 

In [31], the energy consumption of the relay node is refined into transmission energy consumption, RF amplifier loss, encoding/decoding circuit loss, and other idle time transmission energy consumption. In this paper, we focus on the difference in energy consumption of the relay node under the full-duplex and half-duplex relay communication, so the relay communication energy consumption model is set to transmit energy, self-interference energy, and RF amplifier losses. To simplify the modeling process, RF amplifier losses are modeled as linear processes. Therefore, according to the relay energy consumption model proposed in [31], the respective energy consumption of the relay node *di* in half-duplex mode and full-duplex mode can be simplified as:

(12) $$E_{di}^{HD}=\frac{B_{sk}}{R_{di\to DL}^{HD}}\frac{\sqrt{P_{\max}P_{di}^{HD}}}{2\eta_{di}}$$

(13) $$E_{di}^{FD}=\frac{B_{sk}}{R_{di\to DL}^{FD}}\left(\frac{\sqrt{P_{\max}P_{di}^{FD}}}{2\eta_{di}}+P_{di}^{FD}\Delta_{di}\right)$$

where *P*_max_ represents the maximum transmission power of the relay node. *B_sk_* represents the amount of data transmitted by the source user *sk*. $R_{di\to DL}^{HD}$ represents the transmission rate of relay node *di* in the half-duplex mode. $R_{di\to DL}^{FD}$ represents the transmission rate of relay node *di* in the full-duplex mode. The $\eta_{di}\in \left(0.1\right)$ is the utility value of the RF amplifier. $P_{di}^{FD}\Delta_{di}$ represents the energy value consumed by the full-duplex relay node in a unit time to cancel self-interference. The stronger the anti-interference ability of the user equipment, the more energy it consumes.

In general, different relay nodes have different residual energy, and the same relay node has different forwarding intentions for different source nodes. If the residual energy of the relay node is sufficient and the energy used for transmission is only a small part of the residual energy, the quality of the transmission will not be low even if the forwarding willingness is not strong. Similarly, if the residual energy of the relay node is general, but the relay node has a strong willingness to transmit data for the source node, the node can also be considered by the relay node. Above all, this paper proposes the acceptable energy consumption value in combination with the wishing value and residual energy ratio, so the acceptable energy consumption value *E_accept-di_* of the candidate relay node *di* is expressed as:

(14) $$\begin{array}{ll} E_{accept-di}= & \left\{ \begin{array}{ll} E_{self-di}e^{-\left(\sigma \frac{1}{\mu_{di}}+\left(1-\sigma \right)\frac{1}{S_{sk,di}}\right)} & ,\mu_{di}>0\\ 0 & ,\mu_{di}<0 \end{array}\right.\\ where & \mu_{di}=\frac{E_{self-di}-E_{doudi}}{E_{di}} \end{array}$$

where *μ_di_* indicates that the residual energy after consumption accounts for the proportion of the total energy of the relay user *di*. The higher the residual energy ratio *μ_di_*, the smaller the influence on the relay user. *E_di_* indicates the energy value when the device is fully charged. In order to exclude some nodes with low residual energy, the full-duplex energy consumption *E_dou di_* is used to indicate the energy consumption of relay node *di*. *σ* ∈ (0,1) is the weight value between the relay node’s willingness and the relay node’s residual energy ratio. In no particular case, *σ* = 0.5 to adequately reflect the effect of both residual energy ratio and willingness of relay node. $S_{di\to sk}$ indicates the willingness of relay node to transmit data for the source node *sk*, which is measured by a social relationship based on requesting file similarity among users and common friend ratio. According to the literature [32], the willingness $S_{sk,di}$ that the relay node relays data for the source node *sk* can be expressed as:

(15) $$S_{sk,di}=e^{-\theta }\omega_{sk,di}+\left(1-e^{-\theta }\right)F_{sk,di}$$

The request file similarity *ω_sk,di_* and the shared common friend ratio *F_sk,di_* are calculated according to the literature [30], and *θ* represents the density of users in the cell.

Based on acceptable energy consumption value and the energy consumption in the full-duplex and half-duplex mode, the relay transmission mode $M_{di\to sk}$ of any candidate relay node *di* can be expressed as:

(16) $$\begin{array}{l} M_{di\to sk}=\left\{ \begin{array}{l} 0,\;E_{di}^{HD}>E_{accept-di}\;and\;E_{di}^{FD}>E_{accept-di}\\ 1,\;E_{di}^{HD}\leq E_{accept-di}\;and\;E_{di}^{FD}>E_{accept-di}\\ 2,\;E_{di}^{HD}\leq E_{accept-di}\;and\;E_{di}^{FD}\leq E_{accept-di} \end{array}\right.,\forall di\in {\mathbb{Z}}_{sk} \end{array}$$

where $M_{di\to sk}=0$ indicates that the relay node cannot relay for the source node. $M_{di\to sk}=1$ expresses that the relay node *di* can only provide half-duplex relay communication, and it is not will to full-duplex relay communication. $M_{di\to sk}=2$ indicates that the candidate relay node *di* can act as both a half-duplex relay node and a full-duplex relay node.

The acceptable energy consumption value *E_accept-di_* indicates the maximum energy consumption value that the candidate relay node can provide for the source node *sk*. The relay transmission mode determined by *E_accept-di_* can ensure that the consumed energy in relay communication has little influence on the relay node itself. In addition, it can also eliminate the nodes with insufficient power or low wish in candidate relay nodes set **Z***_sk_*. Under normal circumstances, the nodes with insufficient power or low wish are prone to link breaks, so excluding such nodes can effectively improve the stability of transmission.

Since $M_{di\to sk}$ represents the relay mode of the candidate relay node *di*, the HD relay transmission rate and FD relay transmission rate calculated according to (7) and (11) are limited by $M_{di\to sk}$. When the $M_{di\to sk}=0$, it indicates that the candidate relay node *di* cannot relay data for the source node, then $R_{sum\to di}^{HD}=0$ and $R_{sum\to di}^{FD}=0$; $M_{di\to sk}=1$ represents that the candidate relay node *di* can only provide HD relay mode for the source node, then $R_{sum\to di}^{FD}=0$. The relay nodes in the candidate relay set **Z***_sk_* with the zero transmission rate of both the full-duplex mode and the half-duplex mode are excluded, and the remaining relay nodes are stored in $Z_{sk}^{\prime }$.

## 5. Trust Metrics

In Section 4, the physical layer of the candidate relay nodes is considered. It can ensure that the transmission quality of the candidate relay nodes is higher than a minimum threshold. In addition, the acceptable energy consumption of relay node is proposed according to the residual energy ratio and the social relationship between source node and relay node. To ensure the transmission quality of relay communication, the mode of relay transmission and the capability of relay node are determined by acceptable energy consumption. Nevertheless, the transmission reliability of relay nodes is still questionable. In D2D relay communication, relay nodes that intentionally lose the source node’s data or forcibly interrupt the D2D relay communication are called malicious nodes. If a malicious node is selected as a relay node to transmit medical data, it is very likely that important medical data cannot be transmitted to the edge medical node, thereby causing great impact on the source node. However, such malicious nodes are difficult to discover through the transmission quality of the physical layer at this moment. Their discovery needs to explore the long-term social attributes of each relay node. Therefore, this section focuses on explore the long-term social attributes of each relay node and defines the trust degree of each relay node so as to improve relay communication reliability.

The trust metrics are divided into direct trust and indirect trust. The direct trust demonstrates the reliability of the actual transmission of relay nodes in the past. The indirect trust indicates the possibility that the source node and the relay node can direct communicate with each other. The stronger the possibility, the higher the degree of mutual dependence, and the reliability of one of them as a relay node to transmit data will be higher. The trust metric of relay nodes can be better evaluated by combining the direct trust and indirect trust.

### 5.1. Direct Trust

Since direct trust indicates the reliability of the actual transmission, it can be measured as the feedback data accuracy of all user equipment assisted by the relay node. Generally, the quality of the transmission is affected by the geographical location of the user. To improve the poor transmission quality, the system provides relay communication for users. When the system selects a particular relay node to assist the source node, the channel quality and transmission stability of the relay node are relatively high. However, some relay nodes suddenly show malicious behavior during relay communication, such as discarding the data of the source user or forcibly interrupting the transmission. In order to reduce the maliciousness of the selected relay node and improve the transmission security, the base station is responsible for storing the data accuracy of each relay communication. Wherein, in the maximum delay time Δ*t_i_* specified by the source node *i*, the data accuracy *L_i.j_* of relay node *j* is defined as:

(17) $$L_{i,j}=\frac{B_{j-\Delta t_{i}}}{B_{i}}$$

*B_i_* indicates the amount of data that the source node itself needs to transmit, and $B_{j-\Delta t_{i}}$ indicates that the destination node receives the data size through the relay node *j* at the maximum delay time Δ*t_i_*. Due to the instability of the wireless channel and the relay transmission rate affected by the wireless channel, the value of Δ*t_i_* is generally large to reduce the possibility of misjudging malicious node. Let *L_th_* ∈ [0,1] be the data accuracy threshold and the value of *L_th_* is based on the importance of the data transmitted by the current user. In general, the value of *L_th_* is 0.5. If *L_i,j_* > *L_th_*, it indicates the relay node discarded the data of the source node or forcibly interrupted the transmission.

Let *K_di_* = (*k*1, *k*2, …, *k*n) denote all source nodes set assisted by the relay node *di*, where *n* indicates the number of source nodes. Hence, the assist-trust value *Hd_di_* of relay node *di* can be written as:

(18) $$Hd_{di}=\frac{\sum_{i=1}^{n}\frac{\sum_{a=1}^{m_{i}}L_{ki,di-a}}{m_{i}}}{n},\forall L_{ki,di-a}\geq L_{th}$$

where *m_i_* is the assist frequency of source node *ki*. The *L_ki,di-a_* indicates the data accuracy sent by the source user *ki* after transmitting data through the relay node *di* during the *a*-th relay transmission. The one-time data accuracy in relay communication is calculated based on Equation (17). However, most malicious nodes show indirect maliciousness, that is, relay nodes only show maliciousness once or twice in *m* times relays communication. In order to exclude malicious nodes as much as possible, when $\exists L_{ki,di-a}<L_{th}$, *Hd_di_* = 0. Since data accuracy represents the ratio between the forwarded data size and the send data size, the accuracy of the node as actual relay node is not vary greatly in different relay communications after excluding malicious relay nodes. Therefore, the assist-trust value in Equation (18) is calculated by the average value of the data accuracy.

### 5.2. Indirect Trust

The indirect trust is expressed as the possibility of mutual transmission between the source node and the candidate relay node. Generally, if the social relationship among users is relatively strong, the possibility of transmission among users is also relatively large. In the D2D communication mode, the distance among two users is less than the maximum distance of D2D communication for a long time, and the social relationship strength among users is measured by the similarity of the request files and the number of shared friends. When the social relationship is stronger, the possibility of file sharing among users is very large through D2D communication. In this case, if the demand node wants another node to assist in transferring the data to the edge node, the another node is likely to provide a high trust for the demand user in order to obtain the need files from the demand node for a long time. Therefore, the possibility of mutual transmission among the source node and the candidate relay node can be further described as relative distance stable value and social relationship strength.

The relative distance stability is expressed as the number of times and the duration, of which distance value between the source user and the relay user is less than the D2D maximum communication distance at different time points. The more times, the more likely D2D communication occurs between the source user and the relay user. The longer the duration time, the stronger the transmission stability among users and the more likely the transmission is successfully completed. If the value of relative distance stability of users is very high, it can be shown that users have a great similarity in their daily routines and they are often in close proximity to some extent. For instance, users on both sides of the same street, rarely meet due to the road structure of the community, but their relative distance is less than the maximum communication distance of D2D for a long time. 

In general, user’s work habits are very regular, and the location that each user visits every day and the time spent at each location are relatively fixed. Therefore, the location where the user stays longer than *t_th_* is marked as the user’s location of interest. By exploring user’s daily location of interest, a set of points of interest for user *ui* can be represented as $P_{ui}=\left\{p_{ui\to 1}^{T_{ui\to 1}},p_{ui\to 2}^{T_{ui\to 2}},\ldots ,p_{ui\to k}^{T_{ui\to k}}\right\}$, where *k* refers to the number of points of interest, $p_{ui\to k}^{T_{ui\to k}}$ indicates the position coordinates and time information of the *k*-th point of interest for user *ui*. The time domain $T_{ui\to k}$ records the average time of user arriving at a certain location of interest and the average leaving time for many months, and $\left\|T_{ui\to k}\right\|$ represents the average dwell time at the *k*-th point of interest. In general $p_{uj\to k}^{T_{ui\to k}}\neq p_{ui\to 2}^{T_{uj\to k}}$, because different two users *ui*, *uj* are interested in different locations. Therefore, the relative distance stability value *X_ui,uj_* for users *ui* and *uj* is expressed as:

(19) $$\begin{array}{ll} X_{ui,uj}= & \sum_{b=1}^{m}\sum_{a=1}^{k}\left(T_{ui\to a}\cap T_{uj\to b}\right)\sigma_{ui,uj\to a,b}\\ Where & \sigma_{ui,uj\to a,b}=\left\{ \begin{array}{l} 0,D_{P_{uj\to b}}^{P_{ui\to a}}>R_{U}\\ 1,D_{P_{uj\to b}}^{P_{ui\to a}}\leq R_{U} \end{array}\right. \end{array}$$

When the $\sigma_{ui,uj\to a,b}=1$, the distance between two users of interested location can meet the distance of D2D direct communication; $T_{ui\to a}\cap T_{uj\to b}$ means to find the same time period between two users *ui, uj*. *m* and *k* represent the number of interesting points for user *ui* and user *uj*, respectively.

By the relative distance stable value, it can be determined whether the position difference of any two users can satisfy the physical condition of D2D communication. In addition, the social relationship based on both file request similarity and the proportion of common friends affects the possibility of generating D2D links. According to Equation (15), the social relationship between the user *i* and the user *j* can be expressed as:

(20) $$S_{i,j}=e^{-\theta }\omega_{i,j}+\left(1-e^{-\theta }\right)F_{i,j}$$

where the request file similarity ω*_i,j_* and the shared common friend ratio *F_i,j_* are calculated according to the literature [30], and *θ* represents the density of users in the cell.

Based on the above Equations (19) and (20), the relative distance stability and social relationship strength between the user *i* and the user *j* are considered respectively, so the indirect trust *HI_j_* of the source node *i* to the relay node *j* can be expressed as:

(21) $$HI_{j}=\frac{X_{i,j}}{\sum_{\forall j\in {\mathbb{Z}}_{i}^{\prime }}X_{i,j}}\left(e^{-\theta }\omega_{i,j}+\left(1-e^{-\theta }\right)F_{i,j}\right)$$

where $\underset{\forall j\in Z_{i}^{\prime }}{\sum }X_{i,j}$ represents the sum of the relative distance stable value between the source node *i* and each relay node of the corresponding candidate set $Z_{i}^{\prime }$. If the value of *HI_j_* is higher, the more likely the D2D link is generated, the value of higher trust would provide to the source node.

In view of the above consideration for the direct trust and indirect trust, the trust *H_di_* of the source node *sk* to the relay node *di* can be measured as:

(22) $$H_{di}=\alpha Hd_{di}+\left(1-\alpha \right)HI_{di},\forall L_{ki,di-a}\geq L_{th}$$

The term *α* ∈ (0,1) is the weight values between the direct trust and the indirect trust. The direct trust is the comprehensive performance of each relay node in the past relay communication, which is the actual performance of the relay nodes in the past, whereas the indirect trust is obtained through prediction. Therefore, direct trust is more realistic and quantitative than indirect trust. In summary, it is more reasonable to set the weight value of direct trust to be higher than the weight value of indirect trust, and in order to balance the influence of indirect trust, the value of *α* is set to 0.7. if $\exists L_{ki,di-a}<L_{th}$, namely the relay node has discarded data of other source nodes, then *Hd_di_* = 0. In order to eliminate malicious nodes as much as possible, direct trust is more important than indirect trust. According to Equation (22), the trust value for each node in the candidate set $Z_{sk}^{\prime }$ can be calculated and put into the set *E_sk_* in turn.

## 6. Relay Selection Mechanism Based on Compromise Factor

In Section 4, the transmission capability and willingness of each relay node in the relay candidate set are considered, and the transmission mode of each relay user is determined. In the previous section, in order to measure the transmission reliability of each relay node, a relay node trust metric was proposed. When users need to transmit medical data through D2D relay communication, they pay more attention to the reliability of relay nodes to ensure that the medical data can be transmitted to the edge medical node securely, that is, users are more willing to choose the most reliable relay node under meeting their own minimum transmission rate. However, the types of medical data are diverse. For the same user, different data types generally have different requirements for the reliability of relay communication., such as environmental data and important disease monitoring data. For different users, the transmission requirements of the same data type on relay nodes are different, such as the vital signs data of healthy users and patients with chronic diseases. If all the source nodes simply look for the highest reliability relay node at acceptable transmission rate of source node, it would limits the overall spectrum utilization of the system to some extent. For instance, the health user needs to transmit vital sign data. In this paper, the bandwidth of the relay node multiplexing is the same, so when the trust value takes precedence over the transmission rate, the total spectrum utilization of the system is limited to some extent. Based on this, the compromise factor is proposed to give priority to the spectral efficiency of the system for optimal relay selection under certain conditions.

It is assumed that the user equipment determines all data types importance according to the key monitoring type set by the user. Then, the data importance value set by any source user equipment *sk* is represented as ${Ι}_{sk}=\left\{\rho_{1},\rho_{2},\ldots \rho_{n}\right\}$ from high to low, in which $\forall \rho_{i}\in \left(0,1\right)$ and *n* represents the sum of all data types of the source node sk. Different source nodes generally have different values of *n*. When source node *sk* transmits data type with degree of importance of $\rho_{i}$, the overall importance of this data type for all data types *A_sk_* can be expressed as:

(23) $$A_{sk}=1-e^{-\frac{\rho_{i}-\rho_{n}}{\rho_{1}-\rho_{n}}}$$

Let $A_{th}\in \left(0,1\right)$ denote the compromise factor threshold. The value is determined according to the overall physical condition of the source user. The patient’s *A_th_* value is generally less than the healthy user. If the value of *A_sk_* is greater than the threshold *A_th_*, it indicates that the current data is more important, and the user wants to prioritize trust maximization. If the value of *A_sk_* is less than the threshold *A_th_*, it indicates that the data type transmitted by the user at this time is not high enough, and the transmission rate can be maximized. The specific relay node selection rules are as follows:

When *A_sk_* < *A_th_*, indicating that the transmitting data type at this time is of low importance, the user can lower the requirement of the trust value and maximize the transmission rate under certain conditions. Therefore, the optimal relay selection condition is:

(24) $$\begin{array}{ll} \underset{di\in {{\mathbb{Z}}^{\prime }}_{sk}}{\arg} & \max R_{di}=\underset{di\in {{\mathbb{Z}}^{\prime }}_{sk}}{\arg}\max\left(R_{sum\to di}^{FD},R_{sum\to di}^{HD}\right)\\ & st:H_{di}>H_{th-sk} \end{array}$$

where $R_{sum\to di}^{FD},R_{sum\to di}^{HD}$ are determined according to the transmission mode $M_{di\to sk}$ of the relay node *di*. *H_th-sk_* is the lowest acceptable trust value of the source node *sk* to ensure that the selected relay node can meet the minimum requirements of the source node. Since the trust value required by the source node is larger with increasing data importance, the minimum acceptable trust value should be proportional to the importance of the data. Thus, the minimum acceptable trust value *H_th-sk_* is denoted as:

(25) $$H_{th-sk}=\frac{\rho_{i}-\rho_{n}}{\rho_{1}-\rho_{n}}H_{\max}$$

where *H*_max_ represents the maximum trust value provided to the source node *sk* among all relay nodes of the set *E_sk_*.

When *A_sk_* < *A_th_*, *H_th-sk_* is proposed to ensure that the reliability of the selected relay node is not too bad. When *H_th-sk_* is exceeded, the relay node with the highest transmission rate can be directly selected without considering the relay node with higher transmission reliability. By maximizing the transmission rate, it is possible to find a full-duplex relay node with a faster transmission rate, thereby increasing system spectrum utilization and reducing base station load.

When *A_sk_* ≥ *A_th_*, it indicates that the privacy of the data type transmitted by the source node *sk* at this moment is very strong. It is more important to ensure that data can be safely transmitted to the edge medical node compared to reducing the system’s load. Therefore, the optimal relay node selection condition is:

(26) $$\begin{array}{ll} \underset{di\in {{\mathbb{Z}}^{\prime }}_{sk}}{\arg} & \max\left(H_{di}\right)\\ & st\;\exists \left(R_{sum\to di}^{FD},R_{sum\to di}^{HD}\right)>R_{sk-th} \end{array}$$

When *A_sk_* ≥ *A_th_*, the relay node with the highest trust value is preferred. *R_sk-th_* indicates the minimum required rate of the source user. The value of *R_sk-th_* is the average rate at which this type of data is transmitted for the source node in a long time. The values of $R_{sum\to di}^{FD}$ and $R_{sum\to di}^{HD}$ are both limited by the acceptable transmission mode $M_{di\to sk}$ of the relay node.

According to the above relay selection rules, this paper proposes a relay selection algorithm based on the compromise factor, as shown in Algorithm 1.

**Algorithm 1.** Relay selection algorithm based on compromise factor
**1.**
**Input:** source node sk, corresponding candidate relay set ${\mathbb{Z}}_{sk}$, the importance of all data sets ${Ι}_{sk}$ for source node sk
**2.**
**Initialize:**$R_{\max}=0$, $H_{\max}=0$
**3**
**Output:** Optimal relay node $u_{opt}$
**4.**
calculate acceptable energy value $E_{accept-di}$ of any relay node di in ${\mathbb{Z}}_{sk}$ according to Formulas (14) and (15)
**5.**
get corresponding relay mode $M_{di\to sk}$ according to acceptable energy value $E_{accept-di}$ and Formulas (14) and (15)
**6.**
**if**$M_{di\to sk}\neq 0$**then**: put di in set ${{\mathbb{Z}}_{sk}}^{\prime }$
**7.**
calculate the overall importance $A_{sk}$ according to Formula (23)
**8.**

**if**
$A_{sk}<A_{th}$
**then:**

**9.**
    **for**
$di\in {{\mathbb{Z}}_{sk}}^{\prime }$
**do**:
**10.**
        get relay communication rate $R_{di}$ subject to corresponding relay mode $M_{di\to sk}$ according to (7) and (11) and trust value $H_{di}$ according to (18), (21), (22)
**11.**
        **if**
$H_{di}>H_{th-sk}$ and $R_{di}>R_{\max}$
**then**: $u_{opt}=di$ and $R_{\max}=R_{di}$
**12.**
  **end for**
**13.**
**else**:
**14.**
    **for**
$di\in {{\mathbb{Z}}_{sk}}^{\prime }$
**do**:
**15.**
        get relay communication rate $R_{di}$ subject to corresponding relay mode $M_{di\to sk}$ according to (7) and (11) and trust value $H_{di}$ according to (18), (21), (22)        **if**
$H_{di}>H_{\max}$ and $R_{di}>R_{sk-th}$
**then**: $u_{opt}=di$ and $H_{\max}=H_{di}$
**16.**
  **end for**
**17.**

**end if**

**18.**

**End**


## 7. Simulation Results and Analysis

This section will evaluate and analyze the performance of the relay selection algorithm based on compromise factor (RSCF) through MATLAB. The simulation environment is considered as a single-cell model, and the geographical location of users in the cell is subject to random distribution. D2D users reuse the uplink resources with cellular users. The channel model considers Rayleigh fading and path loss. Other specific parameters are shown in Table 1.

In order to fully demonstrate the performance of the proposed RSCF algorithm, several different relay selection algorithms and D2D direct communication are compared in the simulation experiment. The comparison algorithms include the Based on Maximum Throughput for Relay Selection Algorithm (MTRS), the Based on Maximum Social Stability for Selection Algorithm (MSRS) and the Random Relay Selection Algorithm (RRS). The MTRS algorithm and the MSRS algorithm are commonly used to select relay nodes in relay communication, while the algorithm proposed in this paper is the optimal selection algorithm of throughput and trust based on data importance. By comparing these above two algorithms, the performance of the proposed RSCF algorithm can be effectively proved. In addition, we also discuss the impact of different compromise factor thresholds and self-interference cancellation coefficients on the performance of the proposed RSCF algorithm. The specific comparison index is the relay communication’s transmission rate and social stability. Where, the social stability is the trust value between source node and relay node.

### 7.1. Effect of Different Parameter Settings on the Performance of the Proposed Algorithm

In the RSCF algorithm, the transmission mode of each relay node is measured according to the acceptable energy consumption of the relay node. In full-duplex relay communication, the self-interference elimination capability of relay nodes will greatly affect the performance of full-duplex relay communication. Thus, we respectively set the self-interference cancellation coefficient to 40 dB, 80 dB and 113 dB in the simulation experiments, and then compare the performance on the various aspects of the proposed RSCF algorithm. Since the compromise factor threshold *A_th_* directly affects the optimization criteria of the proposed RSCF algorithm, this paper also discusses the performance changes of the proposed RSCF algorithm under different compromise factor thresholds. 

**Figure 3:**
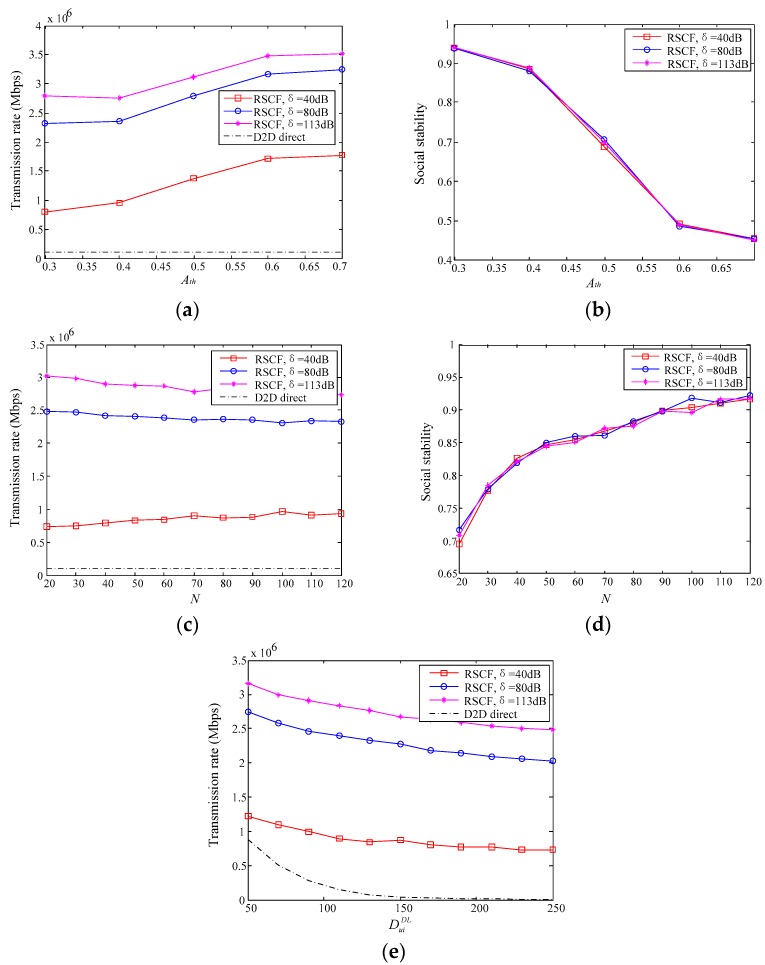
The effect of transmission rate and social stability: (**a**,**b**) Compromise factor *A_th_*; (**c**,**d**) Number of relay candidate nodes N; (**e**) distance $D_{ui}^{DL}$.

Figure 3 shows that the transmission rate is improved greatly as the self-interference cancellation coefficient is increases. Since the larger self-interference cancellation coefficient indicates that the relay node is less susceptible to interference by its own signal, the performance of the full-duplex relay communication is better, thereby the transmission rate is significantly improved. However, the social stability is not affected by the self-interference coefficient. The reason is that social stability expresses the strength of the relationship among users in the social domain, and is not limited by the self-interference coefficient in the physical layer. As shown in Figure 3a,b, with the compromise factor threshold *A_th_* increases, the transmission rate of the D2D relay communication system gradually increases, but the social stability decreases. That is because when the user’s transmission task has been determined, the increase of the compromise factor threshold *A_th_* will cause the proposed RSCF algorithm to be more inclined to find the node that maximizes the transmission rate, so the social stability of the relay communication system is gradually reduced. It is seen from Figure 3a,b that the transmission rate and social stability of D2D relay communication are better as the compromise factor threshold *A_th_* ∈ [0.4,0.6]. When the user pays attention to the transmission rate, the value of *A_th_* is more suitable for 0.6, so that not only the transmission rate of the D2D relay communication is high, but also the reliability of the D2D relay communication can be guaranteed. Conversely, when the user needs to transmit a medical data type with high importance, the value of *A_th_* is 0.4 to guarantee high reliability and meeting the user’s demand for the transmission rate. In the subsequent comparative experiments, the value of *A_th_* is 0.5, which is more universal. From Figure 3b, although the social stability will decrease with the increasing threshold *A_th_*, the social stability of the relay communication link is not very poor under the limit of the minimum trust threshold. Figure 3c–e mainly compare the effect of the number of relay candidate nodes *N* and the distance $D_{ui}^{DL}$ between the user and the edge medical node on the relay transmission rate and social stability. From Figure 3e, with the distance $D_{ui}^{DL}$ from the user to the edge medical node increases, the transmission rate is smaller due to the problem of the path fading.

### 7.2. Performance Comparison of Each Algorithm

**Figure 4:**
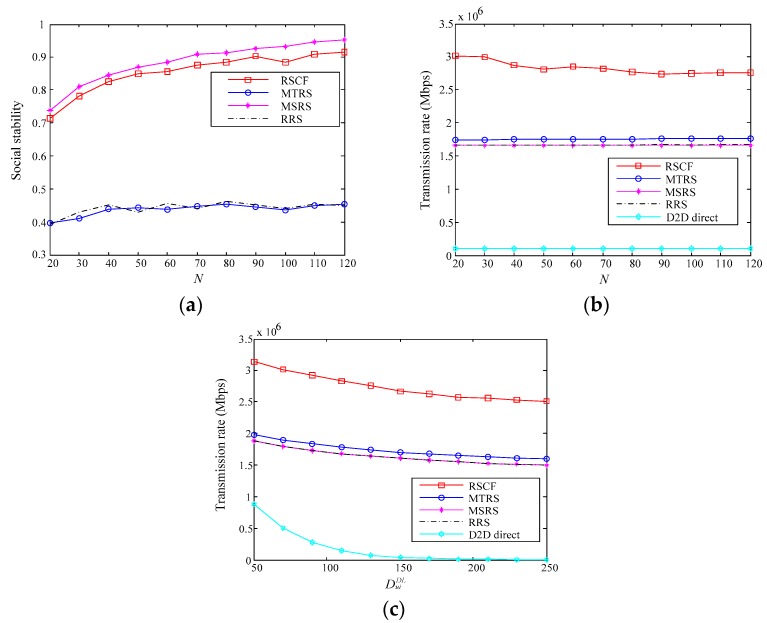
Effect of different parameters on four algorithms: (**a**,**b**) Number of relay candidate nodes *N* for social stability and transmission rate; (**c**) Distance $D_{ui}^{DL}$ of among users to edge medical center node.

In order to fully demonstrate the performance of the proposed RSCF algorithm, this paper compares the performances of the four algorithms under different parameters, as shown in Figure 4.

It can be seen from Figure 4a that the proposed RSCF algorithm is significantly superior to MTSR and RRS in terms of social stability. That is because the source user’s data request importance and compromise factor threshold are considered in the proposed algorithm. When the data request importance is not very high, the RSCF algorithm will focus on increasing the transmission rate. Although the RSCF algorithm is lower than the MSRS algorithm in terms of social stability, the difference between these is small. As shown in Figure 4b,c, the RSCF algorithm is significantly better than the other three algorithms in transmission rate. The main reason is that RSCF algorithm can switch the half-duplex/full-duplex relay communication mode according to the state of the relay node. The transmission rate of RSCF algorithm is improved effectively by using the full-duplex relay mode. As shown in Figure 4b shows that the transmission rates of the other three algorithms tend to be stable with the increasing number of relay candidate nodes *N*. Since the initial value of the number of relay candidate nodes is large and the transmission rate of the relay node is related to the channel quality, and the cell radius in the simulation is small, the relay node with good channel quality can be found at *N* = 20. The transmission rate of the RSCF algorithm proposed in this paper is slightly reduced because the data request importance of the source user will affect whether the relay node selection prioritizes the transmission rate or the trust value. As shown in Figure 4c, with the distance $D_{ui}^{DL}$ from the user to the edge medical node increases, the transmission rate of the four algorithms is relatively reduced due to the problem of the channel fading.

Through the above experiments and analysis, the RSCF algorithm proposed in this paper can flexibly select optimization criteria. The social stability and transmission rate of D2D relay communication system is improved dramatically by the RSCF algorithm.

## 8. Conclusions

In mobile health care, users need to transmit medical data to the edge medical node of the cell through smart devices. In light of the fact that some users are far away from the edge medical node, D2D two-hop relay communication can be used to improve the transmission performance. However, the quality of the D2D relay transmission is affected by the social relationship between the relay node and the source node and the relay node’s residual energy. Especially in mobile health care, the reliability of the selected relay nodes must be good. Thus, in this paper, the acceptable energy consumption value of relay nodes is firstly proposed at the physical domain, and the half-duplex/full-duplex transmission mode of each relay nodes is considered, and the relay nodes with poor energy are eliminated. Then, the trust metric of the relay nodes is proposed to measure the social reliability of each relay link. Through the comprehensive measurement of the physical domain and the social domain, the transmission performance of each relay node can be obtained. Finally, based on the varying importance of medical data, this paper proposes a relay selection algorithm based on the compromise factor (RSCF). With the help of the RSCF algorithm, the low-importance data can increase the system throughput by reducing the requirement of the trust, further increasing the system’s spectrum utilization. Finally, the simulation results show that the RSCF algorithm proposed in this paper is superior to other algorithms in terms of transmission rate. Although the reliability of selected relay nodes is affected by compromise factor threshold, the reliability of selected relay nodes is still higher compared with other algorithms.

## Figures and Tables

**Table 1:** Simulation Parameters.

Parameters	Values
Radius of neighborhood	500 m
Channel bandwidth B	180 KHz
Rayleigh fading	Exponential distribution with a mean of 1
Transmit power of equipment terminals	23 dBm
Users to edge medical center node distance $D_{ui}^{DL}$	[50 m, 250 m]
Number of relay candidate nodes N	[50, 250]
Self-interference cancellation coefficient	{40 dB, 80 dB, 113 dB}
